# Overview of Immunological Responses and Immunomodulation Properties of *Trichuris* sp.: Prospects for Better Understanding Human Trichuriasis

**DOI:** 10.3390/life11030188

**Published:** 2021-02-27

**Authors:** Dewi Masyithah Darlan, Muhammad Fakhrur Rozi, Hemma Yulfi

**Affiliations:** 1 Department of Parasitology, Faculty of Medicine, Universitas Sumatera Utara, Medan 20155, Indonesia; dmasyithah57@gmail.com (D.M.D.); hemma@usu.ac.id (H.Y.); 2 Faculty of Medicine, Universitas Sumatera Utara, Medan 20155, Indonesia

**Keywords:** antigen, innate lymphoid cells 2 (ILC2s), Th1, Th2, interleukin-13, interleukin-10

## Abstract

*Trichuris* sp. infection has appeared as a pathological burden in the population, but the immunomodulation features could result in an opportunity to discover novel treatments for diseases with prominent inflammatory responses. Regarding the immunological aspects, the innate immune responses against *Trichuris* sp. are also responsible for determining subsequent immune responses, including the activation of innate lymphoid cell type 2 (ILC2s), and encouraging the immune cell polarization of the resistant host phenotype. Nevertheless, this parasite can establish a supportive niche for worm survival and finally avoid host immune interference. *Trichuris* sp. could skew antigen recognition and immune cell activation and proliferation through the generation of specific substances, called excretory/secretory (ESPs) and soluble products (SPs), which mainly mediate its immunomodulation properties. Through this review, we elaborate and discuss innate–adaptive immune responses and immunomodulation aspects, as well as the clinical implications for managing inflammatory-based diseases, such as inflammatory bowel diseases, allergic, sepsis, and other autoimmune diseases.

## 1. Introduction

*Trichuris trichiura*, or human whipworm, is one of the soil-transmitted helminths (STHs) in the phylum of Nematoda. *T. trichiura* is grouped similarly with *Ascaris lumbricoides*, as it involves inseparable risk factors with trichuriasis, but it is phylogenetically distinct [1]. There are more than 70 species in the genus of *Trichuris.*, but only *T. trichiura* infects humans as the definitive host [2]. The infection is generated through the ingestion of an embryonated egg that has been fully developed following 2–3 weeks of soil contact [3]. It hatches in the proximal part of the large intestine or cecum induced by the microbiota in the large intestine via direct and indirect contact with bacterial type 1 fimbriae found in colonic microbiota, previously known for its mannose-sensitive bacterial adherence to the mucosal surface [4,5].

The first larvae, L1, soon grows to L4, completing the full 3 months of its life cycle. Finally, adult worms create a supportive environment in the intestinal crypt [6]. The adult worm is equipped with a modified anterior region, called the bacillary band, which is mostly constituted by stichocytes and a few bacillary glands and cuticular inflations and invades the gut mucosa with minor damage. A collective of stichocytes, which is known as a schistosome, also acts as a house producer of several secretory products, as discussed in the next section [7]. Meanwhile, an anterior-ventral portion of the bacillary band, called “cuticular inflation,” is hypothetically involved in the active transport for the host–parasite interaction. It comprises many mitochondria but has not yet been fully elucidated [8]. Adult whipworm lives by implanting its anterior portion into gut mucosa and produces 2000–10,000 eggs per day [9], meaning that adult worm observation is possible in fecal samples, but colonoscopy findings could be an alternative [10]. However, the double-knob egg is more practical for trichuriasis diagnosis. Previously, morphometric analysis was used to differentiate eggs in varied hosts and species [11,12].

Nevertheless, the different analyses between species not only relies on the morphological features of the egg and worm, but the recent classification system for whipworm mainly relies on this conventional system and cannot explain the significant stratification among species. Furthermore, several studies have also demonstrated cross-transmission with a challenge from distant phylogenetic variants, including *Trichuris* sp. Zoonotic transmission includes *T. vulpis* (dog whipworm) and *T. suis* (pig whipworm) being discovered in human hosts [13]. Besides this, *T suis* has also shown identical arrangements with *T. trichiura* based on phylogenetic analysis. Although morphological differences are disputable, molecularly, it still constitutes different genetic lineages [14,15,16,17,18]. Thus, the extensification for using molecular classification systems is also beneficial for investigating inter-species diversity. Extensification includes the analysis of nuclear internally transcribed spacers (ITS) or mitochondrial *cytochrome c oxidase subunit 1* (*cox1*) in order to determine three main clades among different host species [19,20,21].

Meanwhile, parasite incursion into colonic mucosa induces immune cascade activation to support parasite eradication and expulsion. It is not uncommon that the helminthic species’ complex antigenic structure and its secreted substances appear to be the significant challenge for the immune system, from recognition until eradication. There are still some unanswerable facts, particularly relating to the host-resistant phenotype and the complexity of immune activation against *T. trichiura*. Nevertheless, the presence of several facts showing that the *T. muris* (mice whipworms) antigen cross-reacts against human whipworm could boost research on human trichuriasis [22]. In addition, some *Trichuris* sp. species also bear indistinguishable secreted products with human whipworm, creating a near-identical milieu for the research into human trichuriasis [23,24,25,26].

Based on a prior study, it is notable that *Trichuris* sp. modulates the gastrointestinal (GI) mucosa environment, causing it to be less hostile for worm development and survival. However, abundant types of immune cells and specialized structures produce a unique interaction after the first invasion occurs [27,28]. These immunomodulation mechanisms transform the immune system, locally and systemically, in combination to support the livelihood of whipworm. Excretory-secretory (ESPs) and soluble products (SPs) are responsible for immunomodulation features, comprising several protein based-substances and miRNA [29]. It is predicted that dozens of ESPs and SPs are secreted throughout the infection process, but only a few have certain functions. Based on genomic analysis, it was found that more than 50% of *T. muris* protein is homologous with the mammalian origin of exosomes. Gene ontology (GO) terms regarding the molecular function are mostly categorized into binding, catalytic activity, and structural molecule activity. At the same time, three major GO terms in the biological process refer to cellular, metabolic, and single organism processes, confirming the role of secreted products in modulating the immune process. A total of 14 ESPs containing miRNA candidates and 73 proteins have been extracted from *T. muris* [30]. In addition, ESPs and SPs could also transform gut microbiota and increase the susceptibility to bacterial invasion during chronic infection with *Trichuris* sp. [31].

In this work, we present a scientific investigation into the uncharted pathogenesis and immune response against trichuriasis. The discussion explores the role of immune cells in the early development of infection, antigenic recognition, the involvement of innate lymphoid cells (ILCs), and undisputed pro-worm expulsion cytokine surges. However, immune response against trichuriasis is still unsuccessful in most infected hosts. Understanding the immunomodulation properties of *Trichuris* sp. is another key area of discussion in this review. This will open the horizons into managing other diseases that involve surges of proinflammatory cytokines. Nevertheless, there are still major issues relating to human trichuriasis that are not discussed here. For instance, the lower potency of anti-helminthic drugs against *Trichuris* sp., which presents a challenge for the efficacy of mass drug administration (MDA) programs and therefore the discovery of putative vaccines and drugs based on host–parasite and immune interactions with the help of several advances in technology, including X-ray computed tomography (CT), for examining morphological features [32].

## 2. Basic Immunology Concept against *Trichuris trichiura*

*Trichuris* sp. induces a cascade of immunological responses characterized by hyper-IgE and eosinophil or humoral-mediated responses in the human large intestine [33,34,35]. However, several incomplete pictures relating to the immune response against whipworm and other helminth infections have been presented. Besides, the host–parasite interaction could be confounded by several factors that contribute to the complex immunity process against the parasite, including the presence of additional parasite non-self-antigens, ESPs and SPs, infection burden, T helper polarization, antibody responses, host microbiota changes, and bacterial translocation [31,35,36,37,38,39].

Several factors affecting the immune response are also orchestrated locally and systematically during infection, which were represented through a study using peripheral blood lymphocytes (PBL). PBL played a role as a marker for the immunologic response in mesenteric lymph nodes against *T. trichiura*, and ultimately secreted higher levels of type 2 cytokines, including interleukin (IL-4), IL-5, IL-9, and low levels of interferon gamma (IFNϒ) secretion obtained via peripheral blood samples [40]. In another study, trickle infection appeared as a transforming factor of immunological direction. This mode of infection also modulated the immune response during the experimental study more predominantly with Th1-type cytokine. In contrast, a high burden of infection at a single time prevents a large degree of Th1-type cytokine expression, and it is concurrent with a significant change in the resistant phenotype [41,42,43].

In the subsection below, the basic concept of the immunological response against *Trichuris* is discussed regarding the fate of worm expulsion with a potent Th2 response versus chronic infection dominated by Th1-type cytokine expression.

### 2.1. Innate Immune System Also Determines the Fate of Infection

A recent review explored the substantial role of the GI epithelium as the first structural barrier against pathogens, as it maintains the homeostasis with the work of mucus, intestinal microbiomes, and functional innate immune cell content [44]. Besides this, the gut microbiome, symptomatic diarrhea, and pathogen recognition receptors (PRRs), such as Toll-like receptors, nucleotide-binding domain (NOD)-like receptors (NLRs), or C-type lectin receptors (CLRs), contribute to the immune response to *Trichuris* infection [45]. As a result, this generates a hostile environment for *Trichuris* development and proliferation if effective anti-helminthic immunity is formulated.

Intestinal epithelial cells have been notably enrolled in the immune response against intestinal bacterial and parasitic infection by inundating the gut environment with Th2-type cytokine during the initial phase of infection [23,44,46]. Previous studies have shown that colonic epithelial cells and mesenteric lymph node cells (MLNCs) secrete high levels of interferon (IFN) ϒ with a low expression of Th2 cytokines produced both in resistant and susceptible laboratory-infected mice, suggesting a multistage process for immune activation [47,48].

The involvement of GI tissue in secreting cytokines against parasitic invasion was also evident in another study provoking IL1, IL6, IFNϒ, and TNFα mRNA expression [49]. Consequently, Th1 cytokine expression then upregulates and shifts to Th2-type cytokine for the resistant state of infection. However, immunity timelines against *Trichuris* have not been entirely delineated, since strong Th2 immune responses alone could trigger worm expulsion without B cell involvement [50]. Still, a mixed response (Th1/Th2) requires B cells to effectively expedite expulsion in an IFNϒ-rich environment, denoting the involvement of B cells in amplifying Th2 polarization [37,51]. Moreover, local intestinal antigen-presenting cells (APCs) could produce retinoic acid for IL10 production and TGFβ—a cytokine hallmark for priming Th2-type phenotypic responses [52].

**Figure 1:**
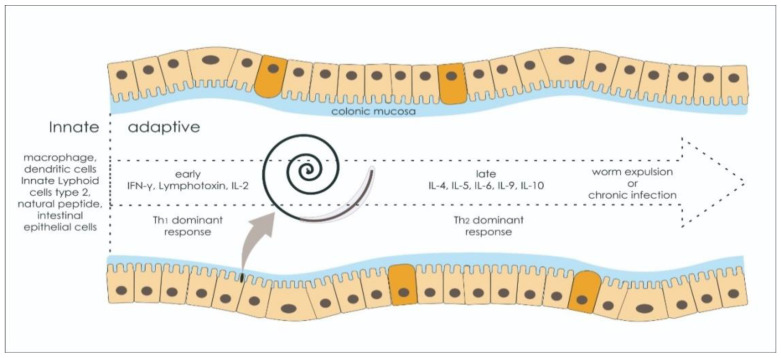
The fate of the immunological response against *Trichuris* sp. infection. There are two critical factors associated with the successful worm expulsion: The host inflammatory genotype and infection burden. The innate immune response must mount Th2-type cytokines.

Classical findings associated with immune activation in *Trichuris* sp. infection have delineated innate and adaptive immune systems as different processes, but recent studies have shown otherwise. The interaction between the two parts of the immune response is responsible for the successful fate of parasite eradication, as it is shown in Figure 1. IL4, IL5, and IL-13 authenticate the activation of Th2 immune response, and these proinflammatory cytokines surge prior to Th2 activation. Intestinal epithelial cells and innate immune cells can upregulate Th2 cytokines via thymic stromal lymphopoietin (TSLP), IL-25, and IL-33 secretion, which are potent inducers for Th2 cytokine expression [53,54,55,56]. Therefore, the innate cellular component could also determine the reactivity and the quality of adaptive immune responses, which could result in a failure during antigenic recognition or cellular induction that consequently reverberates to the priming and proliferation of the adaptive immune system.

In the end, the complex framework between innate and adaptive immune response is a continuation of different immune activation and regulation processes. The amalgamation of functions among immune cells is responsible for leading to worm expulsion by increasing intestinal epithelial cell turnover and mucin secretion. Specifically, IL-4 induce smucin production from enterocytes while IL-13 triggers goblet cell hyperplasia. Both cytokines promote smooth muscle contraction, which ultimately expedites worm expulsion, but cytokine activities might also be independent of the adaptive immune responses [57,58].

### 2.2. Antibody-Dependent Cell-mediated Cytotoxicity (ADCC): Is It Reliable for Trichuriasis?

In another perspective, antibody-dependent cell-mediated cytotoxicity (ADCC) has become an alternative route to eradicate whipworm due to the inability of phagocytic cells to ingest macropathogens [29,59]. The definition of ADCC refers to the immune killing method through the opsonization of antigens using antibodies mediated by cross-linked Fc receptors found in effector cells, such as macrophages, natural killer cells (NK cells), neutrophils, and eosinophils [29]. IgG, IgA, or IgE coat antigens and activate the complement system to finalize the cytotoxic response. In the end, effector cells release granules and lysosomal content and form an identical immunopathology with a “granulomatous appearance” in certain parasitic infections implicated by ADCC, which is highly dependent on sensitized CD4+ T lymphocytes [60,61].

It has long been documented that cytotoxicity represents the essential immune response against *Trichuris* sp. infection, but the role of antibodies has also been notable in some studies. Moreover, some reports have concluded that the role of one of the cytotoxic cell effectors, eosinophil, has been disputed in terms of its protective function during ADCC and its intrinsic function during infection [62,63,64]. The study demonstrated that the worm expulsion or resistance state also continued in the absence of eosinophil and mast cells, while other immune cell subsets affected the regulation [64]. Nevertheless, ADCC might still occur as an innate immune response against helminth larvae, but it could be an inefficient way to generate adult worm expulsion [65].

### 2.3. Adaptive Immune Response: Worm Expulsion versus Chronic Infection

The investigation of the adaptive immune response against *T. trichiura* is incomplete, and several hypotheses and deviant responses have also been proposed during the infection. Early studies divided the terms of “responders,” referring to mice that achieved worm expulsion, and “non-responders,” referring to mice with no immune response against *Trichuris* sp., resulting in chronic infection [66]. Nevertheless, recent studies have disputed the terms because there is still significant immunity established following infection in both groups but inadequate responses among non-responders. Therefore, “resistant” and “susceptible” have been proposed to reflect immune reactivity against *Trichuris* sp. infection. In other words, resistant mice will generate a predominantly Th2-type response, resulting in worm expulsion [67].

Susceptible hosts experience chronic infection and Th1 polarization that is ineffective and even increases immunopathological abnormality [23]. Many studies have supported the fact that Th2-type response predominance against *T. trichiura* quickly results in worm expulsion [36,41,42,47,48,68]. A study discovered that *Schistosoma mansoni* coinfection with *T. muris* caused spontaneous infection resolution [69]. This resistant host phenotype relied on Th2-associated cytokine and antibody isotypes previously produced by the antigenic exposure to the *S. mansoni* egg.

## 3. Innate Lymphoid Cells: A Paucity Not to Be Ignored

Innate lymphoid cells (ILCs) are a part of the innate immune system and contribute to several crucial steps bolstering the immune system against *Trichuris* sp. infection. ILCs arise as the preliminary immune response before the initiation phase of APCs by antigenic exposure to T lymphocytes via the Major Histocompatibility Complex (MHC) II-mediated pathway. Several conditions have notably accelerated the effects of ILCs without the involvement of adaptive immunity [70,71]. ILCs do not express specific lineage markers, as usually demonstrated in other myeloid and lymphoid lineage cells, and comprise two major groups based on the expression of specific transcription factors (TFs), cytotoxic cells (NK cells) and cytotoxic cytokines (ILC1, ILC2, and ILC3 with IL-7Ra+ expression). NK cells and ILC1 are associated with type-1 immune response against an intracellular pathogen, while ILC2, similar to the Th2-type response, acts during parasitic infection, allergic reactions, and tissue remodeling, producing IL-3, IL-5, IL-9, and IL-13, as well as amphiregulin [68].

The identification methods of ILC2s are also defined in the literature, as they are tissue-resident cells [72]. ILC2s are not identifiable with other innate immune cells, but they share similarities with common lymphoid-origin cells by carrying CD45, a common leukocyte antigen. The ILC2 subset has distinct TFs, including a transcription factor of the GATA family, GATA3 [73], which is essential for humoral mediated immunity by Th2 response. However, early ILC2 development and activation also depend on this TF in addition to others, such as BCL11b, RORalfa, and GFi1 [68]. Meanwhile, ILC2 also bears several biologic markers, such as chemoattractant receptor homologous molecule expressed on Th2 cells (CRTH2), MHCII, CD127, CD80, IL-17RB, suppression of tumorigenicity 2 (ST-2), and amphiregulin. ILC2 also has two different subsets, natural ILC2 (nILC2) and inflammatory ILC2 (iILC2), with iILC2 demonstrating proliferative activity during helminth infection [74]. ILC2 also loads a high burden of IL-17RB and killer cell lectin-like receptor G1 (KLRG1), a C-type lectin-like inhibitory receptor with E-cadherin as a ligand which is mostly expressed in NK cells and essential for cell transmigration [74,75].

Innate lymphoid cells 2 (ILC2s) integrate with the adaptive immune system to deliver worm expulsion. During their first handling, antigen recognition hypothetically occurs as the first part of immune triggering for ILC2. Other lymphoid and myeloid lineage cells are involved in the adaptive immune response utilized pattern recognition receptors (PRRs) or non-self-peptides bound to the major histocompatibility complex (MHC) for antigenic recognition, but whether or not this stage also applies during ILC2 first antigenic exposure is still questionable. The bridging of the communication between ILC2s and T helpers ensues with the presence of MHC II-bound ILC2s to exert a type-2 cytokine response against helminth [76]. Nevertheless, three main alarmins (IL25, IL33, and TSLP) secreted from the surrounding environment contribute to ILC2 activation and proliferation following *Trichuris* infection [77,78,79]. Both IL25 and TSLP are secreted by the intestinal epithelial cells, but a specialized epithelial cell called a tuft cell is more abundantly secreted in IL25 production [77,80]. IL33 is another alarmin that is upregulated by the ATP release from necrotic epithelial cells, inducing local mast cells to release IL33. All three alarmins induce IL5, IL13, and amphiregulin as the signature of type-2 immunity against *Trichuris* sp. infection.

In addition, IL25 and IL33 are also secreted by ILC2s, mediating the fibrosis pattern in pulmonary fibrosis and raising questions regarding the stenosis inducer subset of inflammatory bowel diseases (IBD) [81]. Nevertheless, the profibrotic effect of ILC2s is disputed by the fact that ILC2-deficient mice failed to attenuate colitis and fibrosis and had a slower recovery rate compared to ILC2-positive mice with IL13 surges [82]. The presence of cytokine type-2 secreted by ILC2 and TF related to the humoral immune response supports the hypothesis regarding the role of this cell for effective *Trichuris* expulsion.

## 4. Immunomodulation Properties of *Trichuris* sp. and Clinical Implications: Focus on the Role of Excretory/Secretory (ESPs) and Soluble Products (SPs)

Drawing the theoretical background regarding how helminths, particularly *Trichuris* sp., produce chronic infection is not a simple task. During this period, helminth infection deliberately invades the gastrointestinal mucosa and produces a supportive niche for its sustainability. Nevertheless, the infection process only triggers a subtle inflammatory response, frequently flawed Th2 response, or a predominant Th1 immune response. The latest investigation demonstrated one of the remarkable features of *Trichuris* sp.—that it could transform the immune response into favorable states through the secretion of excretory/secretory products (ESPs)—as we elaborate in the following discussion.

ESPs are a group of molecules released from helminths, bearing immunomodulation features during the host–parasite interaction consisting of some uncharacterized substances, proteases, glycolytic enzymes, protease inhibitors, chaperones, miRNA, and antigen homologs or metabolites [30,83,84]. Whipworms organize the substances for survival and modulate the immune response through two primary mechanisms. First, ESPs can manipulate the expression of PRRs or act as cytokine homologs, affecting downstream signaling pathways [85]. Second, immunomodulation features can also trigger several changes in the response to the regulation and polarization of immune cells that could blunt inflammatory responses via anti-inflammatory cytokine secretion, such as IL10 and transforming growth factor β (TGFβ). In a study, the direct effect of the presence of ESPs reduced IL-1β, TNF-α, and NO-2 as secreted products of macrophages in the large intestine [31]. However, whey acidic protein (WAP), as an abundant type of EPs found in *T. muris*, induces induce type-2 immunity that eventually promotes worm expulsion [86].

### 4.1. Secreted Products Modulate Pattern Recognition Receptor (PRR)

ESPs impair the function of PRR, which is an essential part of the innate immune response for antigen recognition. There are several types of PRRs, such as retinoic-acid inducible gene (RIG)-like receptors (RLRs), NLRs, CLRs, and toll-like receptors (TLRs), contained by future antigen-presenting cells (APCs), such as macrophages and dendritic cells (DCs). By modulating the activation of PRRs, the ESPs of *T. suis* were shown to prevent lipopolysaccharide-induced TLR4 sensing in human dendritic cells (DCs) in a study, suppressing downstream signaling pathways [87]. It was also evident that *T. suis* SPs transform macrophages into a more anti-inflammatory phenotype by inhibiting P2RX7, a receptor involved in the stimulation of immune cells such as macrophages, dendritic cells, and lymphocytes, concurrent with reduced IL12B, CCL1, and CXCL9 expression [88]. This interaction reduced the expression of proinflammatory cytokines via Rab7b overexpression, a small GTPase-degrading TLR4 [89,90]. Both pathways related to TLR4 for myeloid differentiation would undoubtedly also be impaired. Reductions mainly involve MyD88-dependent mediated TLR4 responses, finally reducing the level of proinflammatory cytokine genes, reactive oxygen species (ROS), and eicosanoids [89]. ESPs also affected the remaining TLR4 signaling pathway in a TIR-domain-containing adaptor protein-inducing, interferon-β (TRIF)-dependent manner, by reducing IFN α/β production, finally resulting in the scarcity of expression of type I IFNs [91]. *Retinoic acid*-inducible gene (RIG)-I-like receptors also disintegrate following ESP administration, downregulating several essential signaling proteins such as lrf7, Ddx60, and Dhx58 [92]. Thus, there is a decreased downstream signaling for proinflammatory cytokines, and the threats for type-I IFN production become more prominent [93].

Impairing TLR4 activation prevents the surge of proinflammatory cytokine secretion, which eventually results in several clinical implications for other conditions. In sepsis, the hyperactivation of TLR4 and TLR2 is concurrent with the overload of systemic inflammation and organ dysfunction and could produce poor outcomes in animal models [94]. *Trichuris* infection prevents TLR4 activation by downgrading its receptor and signaling pathway, thus reducing the repercussions of proinflammatory cytokine upregulation [88]. Additionally, the immunomodulation properties of *Trichuris* could also increase insulin sensitivity [95]. Dietary fatty acids and enteric lipopolysaccharides (LPS) can activate TLR4 and provoke proinflammatory responses to behave as insulin resistance inducers [96,97]. Therefore, preventing TLR4 signal activation could be the novel target to increase insulin sensitivity.

Glycan-based components in *Trichuris* sp. SPs bind the mannose receptor, a CLR, and increase its expression in monocytes and dendritic cells, inducing protein kinase C (PKC) phosphorylation, specifically PKC δ, and shift the monocyte behavior to an anti-inflammatory phenotype [98,99]. Most novel PKC activation implications remain unknown, but CC chemokine receptor (CCR) 2 and lymphocyte function-associated antigen (LFA) 1 expression are upregulated following PKC activation [100,101]. In IBD, a disease characterized by gut physiology resembling *T. muris* infection, breaking mucosal integrity becomes the basis of pathogenesis, which is perpetually insulted by the expression of proinflammatory cytokine and oxidants caused by the PKC downstream signaling pathway [102]. Moreover, PKC inhibitors were found to attenuate tissue injury in a mice model for colitis [103]. The same study also suggested a breakthrough in advancing qualities for managing several autoimmune diseases with T cells and the autoreactivity of monocyte-derived macrophages using *Trichuris* sp.-secreted products, such as multiple sclerosis and IBD [98].

Cytosolic PRR or the inflammasome have demonstrated several pathogen-associated molecular patterns (PAMPs) and damage-associated molecular patterns (DAMPs) [85]. The presence of ESPs and other extracellular vesicles released from *Trichuris* also affects the function of NOD-like receptor protein 3 (NLRP3), a well-known inflammasome that has pivotal roles during the initiation and amplification phases of both the innate and adaptive immune response [104]. *Trichuris* exosomes encourage pro-helminthic immunity by upregulating IL1β and IL18 via the NLRP3-dependent pathway [104,105]. IL18 appears to be a driving force for the different outcomes since it has a diverse function that could initiate a resistant or susceptible type of immune response. IL18 used to be known as IFNϒ-inducing factor (IGIF) and is involved in the vast signaling pathway for Th1 and NK cell activation, but in vivo studies have suggested that IL18 undermines anti-helminth immunity through an IFNϒ-independent pathway. In other studies, NLRP3 activation triggered downstream signaling pathways of the Th1-type response, making the host susceptible to chronic infection [104]. In contrast, infecting NLRP3-deficient mice with helminths augmented early innate immune cell recruitment, eosinophilia, and neutrophilia, as well as type-2 cytokine responses, while the presence of NLRP3 attenuated immunopathological changes in the tissue environment [106].

### 4.2. Secreted Product Skewed Innate Immune System

*Trichuris* ESPs and SPs have also become key to developing the innate immune system by shifting the response of classical (inflammatory type) into nonclassical monocytes after *Trichuris* SPs administration. This type of monocyte has no expression of CCR2 and CD14 but has a higher expression of CX3CR1. A group of proinflammatory cytokines (a marker of classical monocytes), including IL-10, TGFβ, TNFα, IL-6, and ROS, was first secreted during early observation. However, transition occurred following 16 h of SPs treatment with prominent anti-inflammatory cytokine expression, showing that classical monocytes were largely impacted by the presence of SPs [98].

Monocyte hypermotility, mediated by the activity of small Rho GTPases such as Rho, Rac, and Cdc42 on the actin cytoskeleton, was also notable and reduced adhesion to endothelial cells following *Trichuris* ESPs treatment [98]. A high-saturated prostaglandin E2 content of EPs and SPs also manipulated dendritic cells by skewing proinflammatory features by upregulating RAB7B [90]. PGE2 synthesis by *T. suis* was independent of cyclooxygenase activity in the study. SPs modulate DCs through a mechanism that predominantly involves the overexpression of PGE2, although its effect differs based on its concentration and bound receptors [84]. This could resolve inflammation to accommodate immunopathological repair. However, the glycan component of SPs was also found to interact with CLRs in human DC, with the final result of modulating DCs to suppress proinflammatory responses, but this is a concentration-related effect.

### 4.3. Secreted Product Produce Deviant Cytokine Response

ESPs influence proinflammatory cytokine expression. However, higher levels of IL10 and other regulatory cytokines, TGFβ and IL-35, are associated with the administration of *Trichuris* ESPs. This also suggests that this cytokine concoction yields the main immunomodulation properties of ESPs. The regulatory function of IL10 cannot be described solely as pro- or anti-inflammatory cytokines because of the pleiotropic features caused by heterogeneous receptors IL10Rα, IL10Rβ, IL22Rα, and IL28Rα with diverse implications. Nevertheless, IL10 still augments the Th2-type immune response against acute *Trichuris* infection via IL10Rα activation [107]. It also plays a vital role in protecting the intestinal barriers, preventing other bacterial invasions, and constraining the systemic inflammatory response against *Trichuris* infection. Conversely, several significant findings regarding tissue rupture are caused by *Trichuris* invasion promoting bacterial translocation through the lesion. In two separate studies, the response of *T.suis* inoculation was clearly found to lead to macro-pathological changes associated with bacterial infiltration and the suppression of local immunity to the *Trichuris* sp. site of infection [31,108].

In contrast, it was observed that Alzheimer’s transgenic mice infected with *Trichuris* were more vulnerable to suffer from exacerbations caused by neuroinflammation and larger microglia size, suggesting that a systemic response also developed during overwhelming IL10 secretion [109]. Nevertheless, IL10 upregulation following ESP treatment showed positive implications in other pathological conditions, including IBD. IL10-deficient mice developed chronic inflammation, resulting in the significant immunopathology caused by an incessant immune response against normal intestinal flora [110]. Meanwhile, the reduction of airway responsiveness and IgE production dependent on IL10 was also evident in the mice model for allergic disease, showing the immunoregulatory function of IL10 [111]. Concerning this evidence, ESPs ultimately reduces the inflammatory response and its immunopathology by promoting IL10 secretion.

The administration of ESPs also thwarts Th2 immune cell polarization via the direct activity of ESPs modulating IL4 and IL13 expression, which are useful in different pathways for Th2 immune maintenance [112,113]. This recent finding shows that the protein component of ESPs secreted during chronic *T. muris* infection, called p43, acts as a homolog for IL13 receptor α2 (IL13Rα2) and thrombospondin type 1 [113]. This protein binds into the IL13 active site, inhibiting downstream activation, thus resulting in susceptibility and failed worm expulsion. In predominant eosinophilic diseases, IL13 plays a crucial role in eosinophil priming and proliferation, causing more damage to the tissue environment. Since ESP mimics IL13Rα2, it could halt the vicious pathological changes mediated by IL13 activity, such as airway hyperresponsiveness and goblet cell proliferation, as well as mucus secretion. Therefore, it might be beneficial for asthma, atopic dermatitis, and chronic rhinosinusitis with nasal polyps.

## 5. Conclusions

The Th2-type cytokine mainly plays pivotal roles in alleviating the burden of a worm and thus its expulsion. Nevertheless, innate and adaptive immune responses are evidently connected as the reasons for the success of worm expulsion. The roles of the innate immune system are demonstrated by ILC2s that could provoke Th2-type responses before the involvement of adaptive immune responses. Furthermore, ESPs and SPs mediate major *Trichuris* immunomodulation properties and could support pro-helminthic immunity. However, the scientific investigation of this feature has brought several experimental breakthroughs in the clinical resolution of several autoimmune, allergic, and other chronic immunoreactive diseases. Further research is necessary to confirm its effects in the general population.
